# Down-regulation of *RB1* and *TP53* as potential predicting biomarkers for castration-resistant prostate cancer (CRPC): Indonesian retrospective cohort study

**DOI:** 10.1016/j.amsu.2020.11.017

**Published:** 2020-11-10

**Authors:** Indrawarman Soerohardjo, Irianiwati Widodo, Didik Setyo Heriyanto, Andy Zulfiqqar, Sumadi Lukman Anwar

**Affiliations:** a Division of Urology, Department of Surgery, Faculty of Medicine, Public Health and Nursing, Universitas Gadjah Mada/Dr. Sardjito Hospital, Jl. Kesehatan No. 1, Yogyakarta, 55281, Indonesia; b Department of Anatomical Pathology, Faculty of Medicine, Public Health and Nursing, Universitas Gadjah Mada/Dr. Sardjito Hospital, Jl. Kesehatan No. 1, Yogyakarta, 55281, Indonesia; c Division of Surgical Oncology, Department of Surgery, Faculty of Medicine, Public Health and Nursing, Universitas Gadjah Mada/Dr. Sardjito Hospital, Jl. Kesehatan No. 1, Yogyakarta, 55281, Indonesia

**Keywords:** ADT, Prostate cancer, *RB1*, *TP53*, CRPC, Biomarkers

## Abstract

**Introduction:**

Androgen deprivation therapy (ADT) has remained the first line strategy for treatment of advanced prostate cancers. Despite the profound efficacy of ADT in preventing clinical remission, 30–50% of advanced prostate cancer will develop resistance to hormonal deprivation therapy. This study aimed to evaluate the potential role of *RB1* and *TP53* expressions as biomarkers for predicting time to castration-resistant prostate cancer (CRPC).

**Methods:**

The clinical and pathological data of patients with prostate cancer were collected retrospectively from Dr. Sardjito General Hospital, Yogyakarta. Between 2015 and 2019, a total of 36 patients who received castration were included. Expressions of mRNA of *RB1* and *TP53* from primary tumors were quantified using quantitative Real Time Polymerase Chain Reaction (qRT-PCR).

**Results:**

The expressions of mRNA of *RB1* and *TP53* increased in prostate cancer tissues compared to hyperplastic prostates and significantly downregulated in metastatic prostate cancers (*p* < 0.001). Lower mRNA *TP53* expression correlated with shorter time to CRPC among patients treated with ADT (*p* = 0.006). In addition, stratified analysis showed that lower mRNA *RB1* expression was significantly associated with shorter CRPC both in metastatic (*p* = 0.017) and non-metastatic (*p* = 0.001) prostate cancer patients.

**Conclusions:**

Low expression of mRNA of *RB1* and *TP5*3 has been shown to be a potential marker of shorter time to develop CRPC in patients with advanced stages of prostate cancer treated with ADT. Meanwhile, ISUP score >4 were not shown predictive value on time to CRPC.

## Highlights

• Low expression of TP53 predicted shorter time to CRPC in patients received castration therapy.
• Low expression of RB1 predicted shorter time to CRPC although using different cut-point.
• TP53 and RB1 mRNA expression may provide additional predicted values for time to CRPC.

## 1 Introduction

Prostate cancer has emerged as the second most common malignancy in men and the fifth most common malignancy worldwide with annual incidence of 1.3 million in 2018 [2]. Clinical management for patients ranges from active surveillance for less aggressive prostate cancer to surgery, hormonal therapy, and radiotherapy for advanced cancers. Androgen deprivation therapy (ADT) currently remains the primary anti-hormone therapy for treating prostate cancer [2]. Although ADT is very effective, 28% of patients will develop an aggressive form of castrate-resistant prostate cancer (CRPC). In CRPC, the tumors recur and grow independently from of androgen serum levels and have been variously attributed to the upregulation of androgen receptor (AR) due to *AR* gene amplification, de novo *AR* upregulation by tumor cells, as well as gain-of-function mutations that affect AR stability or affinity for ligands [2].

Several molecular mechanisms have been associated with the development of CRPC such as the low or absence of AR protein expression and neuroendocrine differentiation in anaplastic or small cell/neuroendocrine prostate cancer (SCNC) that are also correlated with unfavorable prognosis [3]. Upregulation of the *AR* gene has also been reported in CRPC due to loss of the retinoblastoma tumor suppressor gene (*RB1*) that affects interactions of E2F1 transcription factors and *AR* genes [4]. Loss of *RB1* function in CRPC has been suggested to cause *AR* overexpression mediated by E2F1 as well as *AR*-target gene overexpression [5]. The *RB1* gene is involved in transcriptional regulation of mitotic checkpoint genes and also contributes to prostate cancer progression through modulation of androgen signaling [5]. In addition, compared to *RB1*, *TP53* is often differentially expressed in CRPC and SCNC and both have been reported to be potential prognostic markers. The *TP53* gene is essential to maintain cellular functions including DNA repair, cell cycle arrest, and apoptosis. *TP53* mutations were reported in 6.9% of prostate cancers [6]. The potential roles of *RB1* and *TP53* down-regulation as predicting biomarkers in the transformation to SCNC are not yet fully revealed. Therefore, this study aimed to evaluate the potential roles of *RB1* and *TP53* expressions in patients with prostate cancer who received ADT as the primary therapy to predict the development of CRPC. This study has been performed and reported according to the STOCSS guidelines [1].

## 2 Methods

### 2.1 Patients

In this cohort study, 40 patients from Dr. Sardjito General Hospital Yogyakarta, between 2015 and 2019, who trans-rectal biopsy guided USG and received castration as the single therapy after diagnosed of advanced prostate cancer were enrolled retrospectively and were divided into 20 samples of non-distant metastases and 20 samples with bone metastases at diagnosis. Clinical and demographic data were collected from electronic medical records. We excluded: (i) patients with any ethnicity other than Indonesian, (ii) patients who had received local treatment before castration, and (iii) patients who received other treatment (such as chemotherapy and radiotherapy) before the disease progression. Two patients were excluded due to sudden death not related with prostate cancer, and two patients were excluded due to failure to follow-up. This study received approval from the Universitas Gadjah Mada, Medical and Health Research Ethics Committee (KE/0158/02/2020). The study was reported according the STROCSS Criteria [7].

On this study, all enrolled patient were received ADT as their therapy. The primary end-point of this study was the time to achieve CRPC, which was defined as secondary radiographic or clinical progress of metastases during castration or/and increase of prostate-specific antigen (PSA) values during castration therapy after achieving nadir values. Clinical staging was determined by unified tumor, node, and metastases criteria according to the EAU 2019 guidelines [8], which were determined by digital rectal examination, magnetic resonance imaging, computed tomography, or bone survey. This study conducted in compliance with the latest Helshinki Declaration (ISRCTN registry; http://www.isrctn.com/ISRCTN24834343) [30].

### 2.2 Quantitative Real Time Polymerase Chain Reaction (qRT-PCR)

RNAs were extracted from formalin-fixed and paraffin embedded primary prostate cancer tissues that biopsied Trans-rectally with ultrasonography guided, and two additional benign prostatic hyperplasia (BPH) which were used as references. The corresponding Samples were moved into several aliquots that fixated with paraffin embedding (TEFE) according to manufacturer instruction, Hybrid-RTM Isolation Kit (GeneAll, Seoul-South Korea) was used to extract total RNAs, and NEXproTM qRT-PCR Kit (NextPro, Seoul-South Korea) was used to quantify RB1 and TP53 expressions. After RNA extracted from paraffin block, the absorbance 280/260 nm known varied 15–21 ng/ml. The primer pair sequences used for the quantification were 5′-GACCCAGAAGCCATTGAAATCT (forward) and 5′-GGTGTGCTGGAAAAGGGTCC (reverse) for RB1 with 5′GCGTGTTTGTGCCTGTCCTG (forward) and 5′TGGTTTCTTCTTTGGCTGGG (reverse) for wild type TP53 exon 8. The amplification conditions consisted of an initial denaturation step at 95 °C for 10 min, followed by 40 cycles at 95 °C for 20 s, at 55 °C for 40 s, and at 72 °C for 60 s. An extension was done at 72 °C for 5 min. The q-PCR amplified samples were performed using BiONEERExi cycleTM 96 (BioNEER, Daejeon, South Korea). RB1 and TP53 expressions were determined by the CT (Cycle Threshold) values and were normalized using GADPH as previously described.

### 2.3 Statistical analysis

The cut-off values for this study were defined according to median expression, while the low and high expression levels were defined as expression lower or higher than median values. Statistical significance was determined using a one-way ANOVA test or Kruskal–Wallis test, and Mann–Whitney U tests were used to compare each group. The differences in times to CRPC between patients with differential *RB1* and *TP53* expression levels were calculated with log-rank analysis on SPSS 24.00 (IBM, USA). *P*-values of *<*0.05 were considered as statistically significant. The figures were generated using GraphPad Prism 7.2 (San Diego CA, USA).

## 3 Results

The mean age of the patients in this study was 69.07 ± 8.7 years old. Mean of PSA levels was 141.22 ± 112.28 ng/ml, and patients were classified with ISUP score 5 (47.2%), ISUP score 4 (11.1%) and ISUP score 1 (13.9%). Surgical castration was performed in 44.4% of patients. The mean time to CRPC was 25.7±18.36 months. Comorbidities were found in the majority of patients including dyslipidemia (44.4%) and type 2 diabetes mellitus (T2DM) 36.1% (Table 1).

**Table 1:** Characteristics of patients.

Variables	n (%)
Ages, years (±SD)	69.07 (±8.7)
PSA, mean (± SD)	141.22 (±112.28)
Mean time to CRPC	25.7 18.36
ISUP Groups (%) ⁃ 1 ⁃ 2 ⁃ 3 ⁃ 4 ⁃ 5	5 (13.9%) 4 (11.1%) 1 (2.8%) 9 (25%) 17 (47.2%)
Castration Methods (%) ⁃ Surgical Castration ⁃ Medical Castration	16 (44.4%) 20 (55.6%)
T Staging (%) ⁃ T1a ⁃ T1b ⁃ T1C ⁃ T2a ⁃ T2b ⁃ T2C ⁃ T3C	4 (11.1%) 2 (5.6%) 9 (25%) 2 (5.6%) 10 (27.8%) 7 (19.4%) 2 (5.6%)
N Staging (%) ⁃ Nx ⁃ N0 ⁃ N1	29 (80.6%) 4 (11.1%) 3 (8.4%)
M Staging (%) ⁃ M0 ⁃ M1B	18 (50%) 18 (50%)
Comorbid (%) ⁃ Cerebrovascular ⁃ Dyslipidemia ⁃ ESRD ⁃ T2DM	10 (27.8%) 16 (44.4%) 7 (19.4%) 13 (36.1%)
n: Number of cases; ESRD: End Stage Renal Disease; T2DM: Type 2 Diabetes Mellitus	

The mean time to CRPC on patients categorized ISUP score less than 4 was 33 months (mean 33.4, 95% CI: 23.8–43.0), which was shorter than patients categorized ISUP score 4–5 (mean 25,7, 95% CI: 3.5–18.8). However, no statistical significance was found between the different ISUP scores (Table 1).

**Fig. 1:**
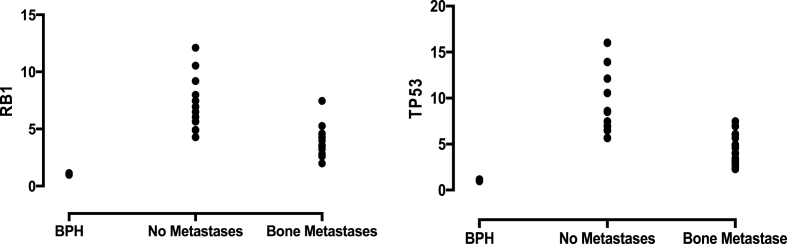
The expressions of RB1 (left) and TP53 (right) on BPH, prostate cancer with No Metastases and Bone Metastases at time of diagnoses *(P* < 0.001).

The expressions of *RB1* and *TP53* were higher in the primary tissues of prostate cancer compared with BPH. *RB1* and *TP53* expression levels were also significantly higher in non-metastatic patients compared to metastatic prostate cancers (Fig. 1) (*p* < 0.0001).

**Fig. 2:**
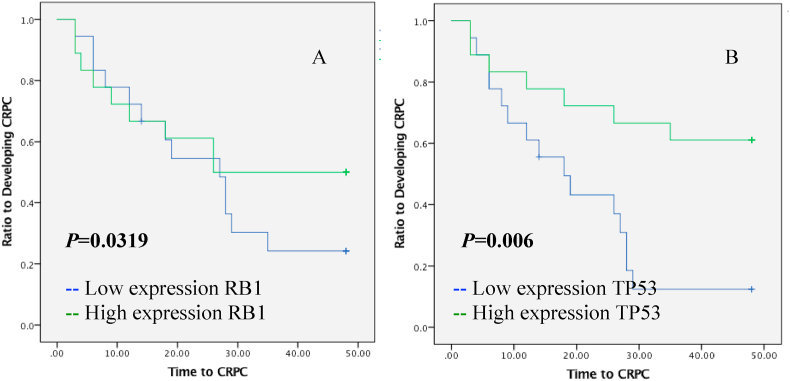
Kaplan-Meier estimates time to CRPC in prostatic cancer patients who received ADT as therapy of prostate cancer for expressions of RB1 (A) and TP53 (B).

**Fig. 3:**
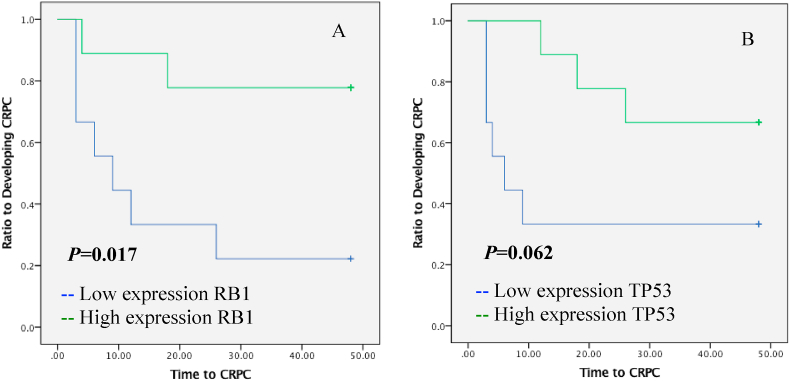
Kaplan-Meier estimates time to CRPC in prostatic cancer patients with bone metastasis treated with ADT as therapy of prostate cancer for the mRNA expressions of RB1 (A) and TP53 (B).

**Fig. 4:**
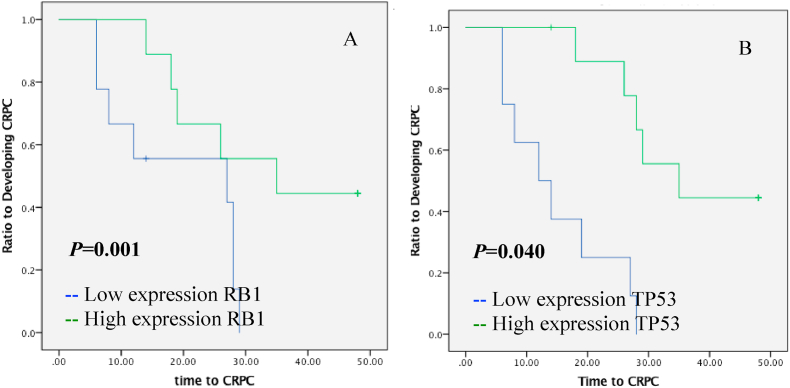
Kaplan-Meier estimates time to CRPC in prostatic cancer patients without bone metastasis treated with ADT as therapy of prostate cancer for the mRNA expressions of RB1 (A) and TP53 (B).

The time to CRPC was not significantly different between patients with lower and higher than median expression of *RB1* (*p* = 0.319, Fig. 2A). In subgroup analysis, in patients with bone metastasis at diagnosis and *RB1* expression lower than median, mean time to CRPC was significantly shorter (mean was 17.6 months; 95% CI: 6.0–29.1) compared to patients with high expression of *RB1* (mean was 39.8, 95% CI: 29.5–50.1), as shown in the Kaplan-Meier survival curve analysis (*p* = 0.017, Fig. 3A). In patients without bone metastasis, patients whose *RB1* expression were low tended to have faster time to CRPC (mean 15 months, 95% CI: 8.9–12.1) than those who presented with high expression (mean 36.4, 95% CI: 29.2–43.7) (*p* = 0.017, Fig. 4A).

The 18 patients with *TP53* expressions below the median had a mean time to CRPC of 20.2 months (95% CI: 13.7–26.6) compared to patients whose expressions were higher than median (mean 35.1 months, 95% CI: 26.9–43.2, Fig. 2B). These results indicate that high expression of *TP5*3 has a significant prognostic value to predict favorable outcome in ADT (*p* = 0.006, Fig. 2B). The subside analysis showed that lower expressions of *TP53* significantly predicted shorter time to CRPC (*p* = 0.040, Fig. 3B) with mean time of CRPC at 19.1 months (95% CI: 12.1–26.157) compared to metastatic patients with higher expression compared to median (mean: 33.8 months, 95% CI: 24.7–42.8). Meanwhile, for patients without metastases, *TP53* levels were not statistically significant for predicting the outcome of ADT (*p=*0.062, Fig. 4B).

## 4 Discussion

In this study, the patients’ age of prostate cancer diagnosis was older and in more advance stages compared to prostate cancers in North America which might reflect the lack of population awareness and cancer promotion as pivotal factors causing delayed on diagnosis [9]. In addition, advanced stages of prostate cancer at diagnosis (ISUP score more than 3) and higher PSA levels at diagnosis were dominant in this study. Contrary to this trend, the majority of European populations reported a low-risk prostate cancer initial diagnosis [10]. Different levels of awareness, health policy and public education on these issues are suggested to be the major factors that contributed to the better diagnosed rate in this region [11].

Metastatic prostate cancer mainly occurs in two ways: patient presents with advanced metastatic cancer at the time of diagnosis and patient experiences recurrence of disease after definitive local therapy [12]. Androgen deprivation axis therapy remains the cornerstone of treatment strategies for managing advanced prostate cancer. Although usually successful as initial therapy, the majority of patients progress with a specific biomarker, clinically or radiographically identified with testosterone level below 50 ng/ml. These patients with this condition are defined as castration resistant prostate cancer (CRPC) [[13], [14], [15]]. Despite the high-caliber arsenal developed for managing CRPC, it remains a very lethal variance of prostate cancer [16].

The androgen based and DNA repair genes are the main focus in surrogate biomarkers of responses to androgen axis therapy [17,18]. These biomarkers can help to guide clinicians in selecting more individualized use of hormonal therapy, and have changed the paradigms of ADT into a more effective precision therapy [19].

ISUP scores has been widely reported as one predicting time to CRPC on patients received ADT (9)(10)(11)(12). However, our study showed that ISUP score 4 and 5 were not shown significant differences. Small number of samples and different ethnicity might underlie the difference with previous (13).

*RB1* has an important role in regulating cell cycle progression, which has clinical impact for treatments focused on targeting cyclin-dependent kinase (CDK) 4/6 inhibitor pathways [20,21]. This strategy is used to target tumors that have lost RB1, which could improve immunological response and the microenvironment and would enhance the outcome of immunotherapy [22,23]. In this study, we found that patients with prostate cancer whose expressions of *RB1* were below median significantly developed CRPC faster compared to patients with expressions higher than the median. The *RB1* expressions were also found to be lower in patients with bone metastases at diagnosis compared to patients with no metastases at diagnosis. These results indicated the cut-off between those arms should be based on the subside analysis, which also demonstrated similar results. The role of *RB1* has been proven valuable to hormonal therapy in an in vitro study [24], and *RB1* depletion was found to promote castration resistant growth and shortened PSA doubling time in vitro models [5]. In clinical settings, *RB1* was also reported to be a predictor in large multicenter cohorts studies, which showed that patients with *RB1* loss have worse outcomes in the first line of ADT [24]. In addition, while not currently feasible, therapy focused on targeting *RB1* mainly exploits the *RB1* loss for therapeutic purposes or reactivating *RB1*'s tumor suppressor function [25].

Loss of *TP53* was also reported to drive AR independent or neuroendocrine tumor phenotypes into prostate cancer [27,28]. In this study, low expression of TP53 was found with significant results to predict shorter time to CRPC only in bone metastatic arms. Meanwhile, the low number of samples may cause the insignificancy in statistical calculation. Concerning the novelty of our research, this is the first study to evaluate *TP53* in prostate cancer. The use of *TP5*3 has been demonstrated in cell line models and mouse models to predict prostate cancer transformation into neuroendocrine cells or CRPC [29]. Additionally, the loss of *TP53* in patients with *RB1* loss worsened the outcome of patients treated with androgen axis therapy [18,30]. The combination loss of TP53 and RB1 in protein levels has been associated with neuroendocrine tumors, and shorter time to responses to ADT and Enzalutamide [31]. However, one preclinical study found that these cancers respond well to a combination of PARP inhibitors and ATR antagonists [32].

The resistance of antiandrogen therapies showed by a variation of histology changed of lineage marker expression. It showed lineage plasticity causes therapeutic resistance. Rb1 loss causes lineage plasticity and metastasis of prostate adenocarcinoma, initiated by Pten mutation. Furthermore, loss of Tp53 and RB1 causes resistance to antiandrogen therapy. Profiling Gene expression purpose of resembling mouse tumors and human PCa NE Variant. Mouse and human tumors show expression of epigenetic reprogramming factors such as Ezh2 and Sox2 increased. Clinically, Ezh2 inhibitors return AR expression and increase AR sensitivity to ADT (29). As long as we concerned, this study is the first cohort studies that evaluated both RB1 and TP53 specifically in Asian population.

The limitation of this study is due to small number of samples that enrolled samples, however, the homogeneity of Race patients also is the strength of this study. Even though time CRPC highly correlated with specific survival of CaP patients, we believe the importance of this data to evaluate the outcome on managing prostate cancer. Therefore, on future direction we need to extend this limitation onto our future direction.

The future direction of this research is in conducting further studies with larger numbers of samples to confirm and validate the findings of this study, since the use of *RB1* and *TP53* expressions on the RNA level has shown promising results. These biomarkers can be used not only as surrogate biomarkers but also possibly as a new option in combination therapy, such as with PARP inhibitors and ATR antagonists. One of the strengths of this study was the samples were all from Indonesian patients, which reduced the possibility of gene heterogeneity. The possibility of a new biomarker approach warrants changing our paradigms in managing this cancer. And we believe, with current numerous studies that avalaible, it's shown that racial and regional approach needs difference strategy to treated CaP.

## 5 Conclusion

This preliminary study suggested that low expressions of *RB1* and *TP53* predicted shorter time to CRPC. Larger studies are recommended to evaluate these biomarkers to change the paradigm into better tailored ADT in patients with prostate cancer.

## Declaration of competing interest

I declare that I do not have any competing interests, especially with the study funder.

## Registration of research studies

In accordance with the Declaration of Helsinki 2013, all research involving human participants has to be registered in a publicly accessible database. Please enter the name of the registry and the unique identifying number (UIN) of your study.

You can register any type of research at http://www.researchregistry.com to obtain your UIN if you have not already registered. This is mandatory for human studies only. Trials and certain observational research can also be registered elsewhere such as: ClinicalTrials.gov or ISRCTN or numerous other registries.

ISRCTN registry; http://www.isrctn.com/

ISRCTN24834343
